# Estimating additive interaction in 2-stage individual participant data meta-analysis

**DOI:** 10.1093/aje/kwae325

**Published:** 2024-08-31

**Authors:** Maartje Basten, Lonneke A van Tuijl, Kuan-Yu Pan, Adriaan W Hoogendoorn, Femke Lamers, Adelita V Ranchor, Joost Dekker, Philipp Frank, Henrike Galenkamp, Mirjam J Knol, Nolwenn Noisel, Yves Payette, Erik R Sund, Aeilko H Zwinderman, Lützen Portengen, Mirjam I Geerlings

**Affiliations:** Department of Health Sciences, Vrije Universiteit Amsterdam, Amsterdam, the Netherlands; Amsterdam Public Health, Mental Health program, Amsterdam, the Netherlands; Amsterdam Public Health, Health Behaviors and Chronic Diseases program, Amsterdam, the Netherlands; Julius Center for Health Sciences and Primary Care, University Medical Center Utrecht and Utrecht University, Utrecht, the Netherlands; University of Groningen, University Medical Center Groningen, Health Psychology Section, Groningen, the Netherlands; Department of Clinical Psychology, Utrecht University, Utrecht, the Netherlands; Amsterdam Public Health, Mental Health program, Amsterdam, the Netherlands; Department of Psychiatry, Amsterdam UMC, Vrije Universiteit Amsterdam, Amsterdam, the Netherlands; Unit of Occupational Medicine, Institute of Environmental Medicine, Karolinska Institutet, Stockholm, Sweden; Amsterdam Public Health, Mental Health program, Amsterdam, the Netherlands; Department of Psychiatry, Amsterdam UMC, Vrije Universiteit Amsterdam, Amsterdam, the Netherlands; Amsterdam Public Health, Mental Health program, Amsterdam, the Netherlands; Department of Psychiatry, Amsterdam UMC, Vrije Universiteit Amsterdam, Amsterdam, the Netherlands; University of Groningen, University Medical Center Groningen, Health Psychology Section, Groningen, the Netherlands; Amsterdam Public Health, Mental Health program, Amsterdam, the Netherlands; Department of Psychiatry, Amsterdam UMC, Vrije Universiteit Amsterdam, Amsterdam, the Netherlands; UCL Brain Sciences, Division of Psychiatry, University College London, London, United Kingdom; Department of Public and Occupational Health, Amsterdam UMC, and Amsterdam Public Health Research Institute, University of Amsterdam, Amsterdam, the Netherlands; Centre for Infectious Disease Control, National Institute for Public Health and the Environment (RIVM), Bilthoven, the Netherlands; CARTaGENE, CHU Sainte-Justine, 3175, Chemin de la Côte-Sainte-Catherine, Montréal, Québec, Canada; CARTaGENE, CHU Sainte-Justine, 3175, Chemin de la Côte-Sainte-Catherine, Montréal, Québec, Canada; Faculty of Nursing and Health Sciences, Nord University, Levanger, Norway; HUNT Research Centre, Department of Public Health and Nursing, Faculty of Medicine and Health Sciences, Norwegian University of Science and Technology (NTNU), Trondheim, Norway; Levanger hospital, Nord-Trøndelag Hospital Trust, Levanger, Norway; Amsterdam UMC, University of Amsterdam, Clinical Epidemiology, Biostatistics and Bioinformatics, Meibergdreef 9, Amsterdam, the Netherlands; Institute for Risk Assessment Sciences, Utrecht University, Utrecht, the Netherlands; Julius Center for Health Sciences and Primary Care, University Medical Center Utrecht and Utrecht University, Utrecht, the Netherlands; Amsterdam UMC, location University of Amsterdam, Department of General Practice, Meibergdreef 9, Amsterdam, the Netherlands; Amsterdam Public Health, Personalized Medicine, and Aging & Later Life, Amsterdam, the Netherlands; Amsterdam Neuroscience, Neurodegeneration, and Mood, Anxiety, Psychosis, Stress, and Sleep, Amsterdam, the Netherlands

**Keywords:** additive interaction, effect-modification, individual participant data meta-analysis, relative excess risk due to interaction (RERI)

## Abstract

Individual participant data (IPD) meta-analysis provides important opportunities to study interaction and effect modification for which individual studies often lack power. While previous meta-analyses have commonly focused on multiplicative interaction, additive interaction holds greater relevance for public health and may in certain contexts better reflect biological interaction. Methodological literature on interaction in IPD meta-analysis does not cover additive interaction for models including binary or time-to-event outcomes. We aimed to describe how the Relative Excess Risk due to Interaction (RERI) and other measures of additive interaction or effect modification can be validly estimated within 2-stage IPD meta-analysis. First, we explain why direct pooling of study-level RERI estimates may lead to invalid results. Next, we propose a 3-step procedure to estimate additive interaction: (1) estimate effects of both exposures and their product term on the outcome within each individual study; (2) pool study-specific estimates using multivariate meta-analysis; (3) estimate an overall RERI and 95% confidence interval based on the pooled effect estimates. We illustrate this procedure by investigating interaction between depression and smoking and risk of smoking-related cancers using data from the PSYchosocial factors and Cancer (PSY-CA) consortium. We discuss implications of this procedure, including the application in meta-analysis based on published data.

## Introduction

Individual participant data (IPD) meta-analyses are increasingly used in epidemiologic research. An IPD meta-analysis is a specific type of systematic review that involves the collection, checking, and re-analysis of the original data for each participant in each study.^1^ One significant strength of IPD meta-analysis is the ability to yield greater statistical power for hypothesis testing compared with smaller-scale individual studies. Compared with meta-analysis based on summary data from previous research, IPD meta-analysis provides a greater control and harmonization in methods used across studies included. In addition, analyses can be conducted that were not previously done, including subgroup analyses or interaction analyses. Hence, utilizing IPD meta-analysis is considered the gold standard method of review.^2^ Individual participant data meta-analysis is particularly interesting for studying interaction and effect modification, as much larger sample sizes are needed to identify interactions or effect modifications than to identify main effects.^3^^-^^5^ In interaction analysis, the main interest is in the combined effect of 2 exposures, while in effect modification the focus is on the possible different effects of 1 exposure within strata based on some other factor.^6^ Many studies that have been designed to examine main effects, such as intervention effects or identification of risk factors, have lacked statistical power to study interaction or effect modification. Combining multiple studies in IPD meta-analysis allows the investigation of, for example, potential interacting risk factors in the development of disease, or identification of subgroups that benefit most from treatment or that are at highest risk for disease.

Several methods on how to study interaction or effect modification within IPD meta-analysis have been developed and compared with respect to power, potential for aggregation bias, and susceptibility to confounding.^7^^-^^12^ These include interaction models for 1-stage IPD meta-analyses, where all data are combined in one dataset that is used to build the statistical model of interest. Alternatively, the meta-analysis of interaction (MAOI) model for 2-stage IPD meta-analysis is a method where the same statistical model is applied to each study and study-specific effect estimates are subsequently pooled in a meta-analysis. The method of choice may depend on data characteristics.^11^^,^^12^ In practice, a 2-stage procedure is often applied when participating studies for various reasons (eg, legal restrictions) are unable to share their individual participant data. In this study, we mainly focus on interaction and effect modification in 2-stage IPD meta-analysis.

Interaction and effect modification can be studied on an additive and multiplicative scale.^6^ Positive *additive interaction* is present if the combined effect of 2 exposures is larger than the *sum* of the individual effects of the 2 exposures. Positive *multiplicative* interaction is present if the combined effect of 2 exposures is larger than the *product* of the individual effects. Additive and multiplicative interaction fall along a continuum: when both exposures increase the risk of the outcome, the threshold for the combined effect to reach additive interaction is lower than the threshold for multiplicative interaction.^13^

Unfortunately, whether multiplicative or additive interaction is being studied is often a matter of convenience, depending on whether the outcome of interest is continuous or binary. Interaction is most often examined by adding a product term of the exposures as an independent variable to the regression model. When performing regression models with a continuous outcome, the effect estimate for interaction is on the additive scale. However, in regression models with a binary or time-to-event outcome (e.g., logistic regression or Cox regression), the effect estimate for interaction is on the multiplicative scale. Current methods on studying interaction in IPD meta-analysis follow the same approach. Although multiplicative interaction is often the default in models with a binary or time-to-event outcome, additive interaction may better fit the objectives of the study.

When statistical interaction is examined to test potential biological interaction, the choice for studying multiplicative or additive interaction should be based on hypothesized underlying biological mechanisms.^14^^,^^15^ When 2 exposures are hypothesized to each reinforce the same biological mechanism or to act at different stages in the same disease process, positive additive interaction may more likely be identified than multiplicative interaction. In addition, the additive scale is preferred from a public health perspective for which absolute risks are more important than relative risks.^6^^,^^14^^,^^15^ Additive interaction indicates, for example, which subgroups are at highest risk for disease or may benefit most from an intervention. By studying interaction on the additive scale, absolute risks due to interaction can be derived. These are informative in number needed to treat to prevent one additional bad outcome or in targeting interventions to subgroups, which may benefit most when resources are limited.

To study additive interaction for binary and time-to-event outcomes, several measures have been developed that can be derived from relative risks (RRs), odds ratios (ORs), or hazard ratios (HRs). The relative excess risk due to interaction (RERI) is most often considered in the literature.^6^ Other measures are the proportion attributable to interaction of the combined effect of 2 exposures (AP) or the synergy index (S).^16^^,^^17^ Thus far, few studies used RERI or other additive interaction measures to study interaction within IPD meta-analyses. In 1-stage IPD meta-analysis, additive interaction measures can be calculated from effect estimates obtained from the analyses on the pooled data.^18^ In 2-stage IPD meta-analyses, different procedures have been used. Some studies calculated additive interaction measures within each study and subsequently pooled study-specific estimates.^19^^-^^21^ Other studies have first pooled the individual effect estimates of the exposures and product term and calculated measures of additive interaction based on these pooled estimates.^22^^,^^23^ There is currently no in-depth discussion in the literature regarding the validity of these procedures.

The aim of this study was to describe how measures of additive interaction and effect modification for binary and time-to-event outcomes can be validly estimated within 2-stage IPD meta-analyses. We propose a procedure to calculate measures of additive interaction based on the pooled effect estimates at stage 2 of the IPD meta-analysis. For simplicity, we will focus on interaction, but our conclusions are also relevant for effect modification. For the same reason, we will focus on the RERI, which is most commonly used, but our approach can also be used to estimate AP or S. We will first explain the RERI and its features in more detail and discuss potential issues related to direct comparisons and pooling of study-level RERI estimates. Second, we provide a 3-step procedure on how to obtain a valid estimate of additive interaction within 2-stage IPD meta-analyses. We illustrate our procedure using an example from the psychosocial factors and cancer (PSY-CA) study.^24^^-^^26^ Finally, we discuss for which situations this procedure can be applied, including meta-analysis based on summary data of previous research.

## Relative excess risk due to interaction (RERI)

The RERI is a measure of additive interaction or effect modification for models with a binary outcome. For 2 dichotomous exposures *A* and *B*, RRs can be calculated for when both exposures are present (*A*+*B*+), exposure *A* is present and *B* is absent (*A*+*B*-), and exposure *B* is present and *A* is absent (*A*-*B*+), with the absence of both exposures (*A*-*B*-) as the reference group. Subsequently, the RERI estimate is calculated as follows:


(1) \begin{equation*} \mathrm{RERI}=\mathrm{RR}_{A+B+}-\mathrm{RR}_{A-B+}-\mathrm{RR}_{A+B-}+1 \end{equation*}

The RERI can range from minus infinity to infinity; RERI = 0 reflects no interaction; RERI > 0 reflects positive interaction; and a RERI < 0 reflects negative interaction (ie, the combined effect of 2 exposures is *smaller* than the sum of the individual effects of the 2 exposures). By multiplying the RERI with the absolute background risk, the absolute risk due to interaction is estimated.^27^ The RERI formula can also be applied to ORs derived from logistic regression models or HRs from models with time-to-event outcomes when ORs and HRs approximate RRs.^6^^,^^28^ A more general formula that can be applied when either one or both exposures are continuous is as follows^6^^,^^27^:


(2) \begin{equation*} \mathrm{RERI}={e}^{\ {\hat{\beta}}_1+{\hat{\beta}}_2+{\hat{\beta}}_3}-{e}^{ {\hat{\beta}}_1}-{e}^{{\hat{\beta}}_2}+1 \end{equation*}

where ${\hat{\beta}}_1$ and ${\hat{\beta}}_2$ refer to the estimated regression coefficients of exposure 1 and exposure 2, and where ${\hat{\beta}}_3$ refers to their product term.

Several methods have been described to calculate a 95% confidence interval (CI), including the Delta method,^29^ 4-by-2 table method,^30^ or bootstrapping. A detailed discussion of these methods for CI estimation is provided by VanderWeele and Knol.^6^

Importantly, RERI but also AP and S should only be applied to exposures that increase the risk of the outcome but not to exposures decreasing the risk (i.e., preventive factors).^31^ Using preventive exposures can give inconsistent results. This is because these measures are calculated from ratio measures (RRs, ORs, or HRs), which have an asymmetric scale: preventive effects are limited between 0 and 1, while risk effects may vary between 1 and infinity. Therefore, preventive factors should be recoded so that the category with the lowest risk is the reference category.^31^

## Comparing and pooling study-level RERI estimates: Potential issues

When additive interaction is examined in 2-stage IPD meta-analysis but also in meta-analysis based on summary data from previous research, this is typically done by calculating estimates of additive interaction for each individual study and subsequent pooling of these estimates.^19^^-^^21^^,^^32^^,^^33^ Here we will discuss potential issues related to direct comparisons and pooling of study-level RERI estimates.

### Inconsistent direction of exposure effects across studies

When performing a 2-stage IPD meta-analysis, the direction of the effect for an exposure may differ across studies: an exposure may be related to an increased risk in study *A*, while a decreased risk may be found in study *B*. As described previously, a RERI estimate can only be validly calculated for exposures that are related to increased risk of the outcome. As such, different reference categories should be used in study *A* and *B* in order to obtain a valid RERI estimate in each study. When changing the reference category for part of the studies, interpretation of RERI will differ across studies and cannot be compared. In this situation, pooling RERI estimates to derive one interpretable effect is impossible.

### Inconsistency with exposure effect estimates due to asymmetry of RERI components

If both exposures are consistently associated with increased risk of the outcome across studies, pooling of study-level RERI estimates might seem valid given its continuous scale and its potential range from minus infinity to infinity. However, the RERI estimate is a function of multiple ratio measures, either formulated in terms of RRs, ORs, or HRs. Due to the asymmetry of these components, the distribution of RERI estimates may deviate significantly from a normal distribution. This complicates direct pooling of RERI estimates across studies using classical methods for meta-analysis. This issue is similar to that when pooling ratio measures like RRs, ORs, and HRs that are usually log transformed to obtain a symmetric scale.^34^ However, a log transformation of RERI is less straightforward, as it is a sum of multiple ratio measures. *Direct pooling of study-level RERI estimates in 2-stage IPD meta-analysis is likely to result in an estimated overall RERI that does not match the pooled effect estimates for both exposures and their product term (i.e., the multiplicative interaction effect).* We illustrate this potential deviation using real-world data in a supplementary data file (Supplementary file, Table S1, Table S2).

## Recommended procedure: calculate overall RERI on pooled effect estimates

To bypass the issues described previously, we propose a procedure to examine additive interaction using the RERI within a 2-stage IPD meta-analysis. This procedure consists of 3 steps:

1. Study-level effect estimation of exposures and their product term on the outcome
2. Pooling effect estimates in multivariate meta-analysis
3. Estimation of overall RERI and 95% CI based on pooled estimates

Step 1 is conducted during stage 1 of the IPD meta-analysis, while steps 2 and 3 are conducted during stage 2. The steps are described into detail here:

### Step 1: Study-level effect estimation

To test interaction for dichotomous and/or continuous exposures, both exposures and their product term are entered as independent variables in the regression model of interest within each study. Alternatively, if both exposures are dichotomous, a categorical variable could be created including 4 categories: both exposures present (*A*+*B*+), only exposure *A* (*A*+*B*-), only exposure *B* (*A*-*B*+), and absence of both exposures (*A*-*B*-). This variable could be entered in the regression model, with the category of (*A*-*B*-) as reference group. Next, covariates can be entered into the model to control for potential confounding in the associations between both exposures and the outcome. From this model, regression coefficients of the exposures and the product term, together with their standard errors, variances, and covariances need to be extracted as input for stage 2.

### Step 2: Pooling of effect estimates

After interaction analyses are performed in each study, effect estimates for both exposures and the product term of all studies are pooled at stage 2. Effect estimates can be pooled in a fixed-effects model, assuming that the true effect is the same in each study, or in a random-effects model, which allows for between-study heterogeneity in the true effect. In most meta-analyses, univariate meta-analysis models are used, pooling one effect estimate at a time. Alternatively, in multivariate meta-analysis, multiple effect estimates are pooled at the same time, taking into account correlations between effects. We recommend using multivariate meta-analyses to pool the effect estimates of the exposures and the product term and their variances and covariances all at once. By using this method, the correlations between the regression coefficients of the exposures and their product term are accounted for. Moreover, pooled variances and covariances of effects are automatically estimated, which are required for estimation of the 95% CI of the overall RERI. More detailed information on using multivariate meta-analyses can be found elsewhere.^35^

### Step 3: Overall RERI estimation

The pooled effect estimates from the multivariate meta-analysis can then be entered in the RERI formula (1) or (2) to obtain an estimate for additive interaction. The 95% CI of the overall RERI can be estimated based on the pooled variances and covariances of the regression coefficients.

As described earlier, RERI should only be applied to exposures increasing the risk of the outcome but not to exposures decreasing the risk. This means that the *pooled* regression coefficients for both exposures should be positive. If the pooled regression coefficient for one of the exposure variables is negative, it is invalid to use this regression coefficient to estimate an overall RERI. This can be solved by reversely coding the preventive exposure variable in *all studies*. Subsequently, steps 1 and 2 have to be repeated and the overall RERI can be calculated. Alternatively, it is also possible to reverse in all studies the regression coefficients of the preventive exposure and the product term and their covariances with the second exposure. This reversal should be incorporated in interpreting the direction of the RERI estimate.

## Example: depression, smoking, and smoking-related cancers in PSY-CA study

We will now illustrate the proposed 3-step procedure with an example from the PSYchosocial factors and Cancer (PSY-CA) study, including a time-to-event outcome. The PSY-CA consortium, involving 18 prospective cohort studies, was established to perform IPD meta-analyses on the association of depression, anxiety, and other psychosocial factors with cancer incidence.^24^^-^^26^ A detailed description of the PSY-CA study, including study protocol and power calculations, has been published previously.^24^ Of note, the power analysis in PSY-CA concerned univariate instead of multivariate meta-analysis and multiplicative instead of additive interaction was considered. Notice that our power calculation is conservative, since power is larger for multivariate meta-analysis than for univariate meta-analysis^36^ and larger for testing additive interaction than for multiplicative interaction.^5^ In this example, we examined potential interaction between depression and smoking at baseline on smoking-related cancers incidence during follow-up, which involves an adapted analysis previously published in Basten et al.^26^ We used IPD of 6 cohorts: CARTaGENE,^37^^-^^39^ the English Longitudinal Study of Aging (ELSA),^40^ the Healthy Life in an Urban Setting (HELIUS) study,^41^^,^^42^ HUNT 2,^43^ Lifelines, ^44^^,^^45^ and the Netherlands Study of Depression and Anxiety (NESDA).^46^ To ease interpretation of results, dichotomous measures of depression and smoking were used. Depression (yes/no) was based on clinical interviews or, if not available, on symptom questionnaires using validated clinical cut-offs. Smoking was defined as currently smoking (yes/no). Cancer cases during follow-up, including cancer type and date of diagnosis, were identified through linkage to national or regional cancer registries. This analysis included a total of 252 686 participants, 7 401 incident smoking-related cancer diagnoses, and 2 536 124 person years of follow-up. All analyses were performed using R Statistical Software.^47^

In step 1, an interaction model using Cox regression analysis was estimated in each cohort study. We included depression, smoking, and the product term of depression and smoking as independent variables into the model and smoking-related cancer incidence as dependent variable. Entry age (age at baseline) and exit age (age at diagnosis, death, loss to follow-up, or study end) were entered in the model as time variables. The model was adjusted for potential confounders of the associations between both depression and smoking with cancer incidence. For simplicity, we present a basic model adjusted for confounders that were available in all cohorts: sex, education, country of birth, and birth year. Regression coefficients for depression, smoking and the product term in each cohort study are presented in Table 1.

**Table 1:** Regression coefficients of depression, smoking, and their interaction on smoking-related cancer incidence, an example from the PSY-CA consortium.

**Cohorts**	**PY (events)**	**Depression B (95% CI)**	**Smoking B (95% CI)**	**Product term B (95% CI)**
CARTaGENE	236 496 (1 334)	0.28 (−0.02 to 0.58)	0.59 (0.46 to 0.72)	0.02 (−0.44 to 0.48)
ELSA	106 877 (705)	0.23 (0.03 to 0.44)	0.87 (0.67 to 1.07)	−0.08 (−0.43 to 0.28)
HELIUS	100 986 (142)	0.09 (−0.55 to 0.73)	0.76 (0.38 to 1.14)	0.19 (−0.72 to 1.11)
HUNT 2	1 038 540 (3 607)	0.15 (−0.09 to 0.38)	0.76 (0.69 to 0.83)	−0.07 (−0.41 to 0.26)
Lifelines	1 023 663 (1 528)	0.14 (−0.23 to 0.51)	0.64 (0.52 to 0.76)	0.00 (−0.55 to 0.56)
NESDA	29 561 (85)	0.09 (−0.61 to 0.79)	0.95 (0.38 to 1.51)	0.18 (−0.72 to 1.08)
Pooled estimates	2 536 124 (7 01)	0.18 (0.06 to 0.31)	0.72 (0.62 to 0.81)	0.01 (−0.18 to 0.20)

Abbreviation: PY, person years. Regression coefficients (B) are derived from Cox regression models within each cohort. Adapted analysis previously published in Basten et al.^26^

In step 2, results from all participating cohorts were pooled using random-effects multivariate meta-analysis. Analyses were performed using R package “mvmeta”.^48^ We used a random-effects model to account for potential varying effects across cohorts, as cohorts within PSY-CA included both population-based and clinical samples and varied substantially in age and time of follow-up. The regression coefficients, variances, and covariances of depression, smoking, and the product term of each cohort were entered in the multivariate meta-analysis. The pooled effects showed that depression (B= 0.18; 95% CI, 0.06-0.31; HR= 1.20; 95% CI, 1.06-1.36) and smoking (B= 0.72; 95% CI, 0.62-0.81; HR= 2.05; 95% CI, 1.86-2.25) were both associated with an increased risk of smoking-related cancer (Table 1). The product term, representing multiplicative interaction, showed that the combined effect of depression and smoking was not higher than *the product* of the individual effects (B_product_= 0.01; 95% CI, −0.18 to 0.20; HR= 1.01; 95% CI, 0.84-1.22).

As both depression and smoking were associated with an increased risk (ie, no preventive effects), we proceeded with step 3. We entered the pooled effect estimates (Table 1) in the RERI formula (2): RERI = e^(0.18 + 0.72 + 0.01)^ – e^(0.18)^ – e^(0.72)^ + 1 = 0.23. The overall RERI was larger than 0, suggesting that the combined effect of depression and smoking is larger than the sum of the individual effects. The hazard ratio of having depression and being a smoker is 0.23 more than if there were no interaction between depression and smoking. We calculated the 95% CI based on the pooled variances and covariances using the delta method. This was done using the function “deltamethod” in R package “msm”.^49^ This resulted in a 95% CI ranging from −0.14 to 0.61, indicating that there is no strong evidence for the presence of an interaction effect and a large interaction effect seems unlikely.

This example included 2 categorical exposures. We could have also tested interaction using a categorical independent variable including 4 categories (2 [yes/no depression] ×2 [yes/no smoking]) in step 1 and calculate an overall RERI at step 3, using formula 1. This procedure would have resulted in the same overall RERI estimate.

## Discussion

Individual participant data meta-analyses provide important opportunities to study interaction and effect modification for which individual studies often lack power. This article describes a procedure to study additive interaction for binary and time-to-event outcomes within 2-stage IPD meta-analyses. Since additive interaction may more closely reflect hypotheses on biological interaction and is more relevant from a public health perspective, this article provides an important addition to previous methodological studies on interaction within IPD meta-analysis.^7^^-^^12^ The procedure was illustrated using an example from the PSY-CA study. We used this procedure previously to examine whether various psychosocial factors, including depression and anxiety, interact with or modify the effects of health behaviors, such as smoking and alcohol use, in relation to cancer incidence.^26^ To date, few previous studies have used a similar procedure as presented in this article.^23^

This article describes a procedure to study additive interaction using the RERI in 2-stage IPD meta-analysis, but this procedure has wider implications. First, the same procedure can also be applied for testing effect modification, that is when the effect of only 1 exposure on the outcome is of interest across subgroups. A main difference in testing effect modification is that only confounders for the exposure are included but not for the effect modifier.^50^ Second, the proposed procedure can also be used to estimate other measures of additive interaction or effect modification, including the attributable proportion due to interaction (AP) and the synergy index (S). Overall estimates of AP and S can be calculated based on the same pooled regression coefficients of the exposures and their product term in step 3. Third, the example from the PSY-CA project involved 2 dichotomous exposures and time-to-event data, which were analyzed using Cox regression. The procedure is also applicable to models including 1 or more continuous exposures and to logistic regression models. Fourth, similar steps may also be used to study additive interaction in meta-analysis based on published studies. If individual studies provide effect estimates for the exposures and the product term, these can be pooled and subsequently used to calculate an overall RERI estimate. However, a major limitation with published articles is that variances and covariances of the effect estimates, which are needed for estimating a multivariate meta-analysis and the final CI of the overall RERI, are often not reported. Possible solutions to deal with missing variances and covariances in multivariate meta-analysis have been described elsewhere.^35^ Finally, additive interaction may also be estimated in 1-stage IPD meta-analysis. The effect estimates for the exposures and their product term obtained in 1-stage IPD meta-analysis can be used to estimate the RERI or other measures of additive interaction. The practical implications of applying one-stage or 2-stage IPD meta-analysis are described elsewhere.^8^^,^^12^

The choice for studying multiplicative or additive interaction should depend on the objectives of the study and not be just a matter of convenience. Knol and VanderWeele provide recommendations for presenting analyses of effect modification and interaction.^50^ They propose to present measures of interaction on additive and multiplicative scales. In this way, sufficient information is provided to the reader to interpret interaction on whatever scale is preferred.

Individual participant data meta-analyses are well-suited to study interaction and effect modification. The current article describes a procedure to study additive interaction for binary and time-to-event outcomes within 2-stage IPD meta-analysis, illustrated with an empirical example. Our procedure will hopefully encourage researchers to apply additive interaction analysis within ongoing IPD meta-analyses projects. Furthermore, this procedure may encourage researchers of smaller studies who aim to study interaction to set up an IPD meta-analysis project.

## Acknowledgments

We thank the whole CARTaGENE team (https://cartagene.qc.ca/en/about), represented by authors N.N. and Y.P., for their contribution. The Trøndelag Health Study (HUNT) is a collaboration between HUNT Research Centre (Faculty of Medicine and Health Sciences, Norwegian University of Science and Technology NTNU), Trøndelag County Council, Central Norway Regional Health Authority, and the Norwegian Institute of Public Health. The HELIUS study is conducted by the Amsterdam University Medical Centers, location AMC, and the Public Health Service of Amsterdam. Both organizations provided core support for HELIUS. The HELIUS study is also funded by the Dutch Heart Foundation, the Netherlands Organization for Health Research and Development (ZonMw), the European Union (FP-7), and the European Fund for the Integration of non-EU immigrants (EIF). Lifelines is a multidisciplinary prospective population-based cohort study examining in a unique 3-generation design of the health and health-related behaviors of 167 729 persons living in the north of the Netherlands. It employs a broad range of investigative procedures in assessing the biomedical, sociodemographic, behavioral, physical, and psychological factors that contribute to the health and disease of the general population, with a special focus on multimorbidity and complex genetics. The Lifelines initiative has been made possible by subsidy from the Dutch Ministry of Health, Welfare and Sport, the Dutch Ministry of Economic Affairs, the University Medical Center Groningen (UMCG), Groningen University and the Provinces in the North of the Netherlands (Drenthe, Friesland, Groningen).

## Supplementary Material

Web_Material_kwae325

## Data Availability

The data that support the findings of this study are owned by participating cohort studies. Data may be shared upon reasonable request at each cohort depending on cohort-specific regulations. Further information is available from the corresponding author upon request.
