# Supplementary material for: Estimating additive interaction in 2-stage individual participant data meta-analysis
Source: Am J Epidemiol. 2024 Aug 31;194(6):1661–7. doi: 10.1093/aje/kwae325 (PMC12448865; doi:10.1093/aje/kwae325)
Supplement: Web_Material_kwae325 [file web_material_kwae325.pdf]

Article: Estimating additive interaction in two-stage individual participant data meta-analysis.

Supplementary data file

Table of contents

Supplementary file: Comparing the results of pooling study-specific risk estimates versus pooling study-specific RERIs to estimate additive interaction in two-stage IPD meta-analysis: a real world example. 2-3

Table S1: Study-level odds ratios (OR) and RERI for the risk of venous thrombosis associated with factor V Leiden (*F*), hyperhomocysteinemia (*H*) and the combination of both factors, derived from the meta-analysis of Keijzer et al (2007). 4

Table S2: Overall RERI based on pooling study-level RERI estimates and overall RERI based on pooled effect estimates. 5

Comparing the results of pooling study-specific risk estimates versus pooling study-specific RERIs to estimate additive interaction in two-stage IPD meta-analysis: a real world example.

Our proposed procedure is based on two premises. First, meta-analysis of study-level effect estimates can be used to estimate true effects at the population level. Second, the RERI is a good derived measure to quantify additive interaction implied by the effect estimates for individual exposures and their product term. Based on these premises, the aim is to obtain a RERI estimate that fits with the estimated effects of the individual exposures and their product term in the population. In our proposed procedure this is the case by definition: the pooled effect estimates for the exposures and their product term are used to calculate the RERI. In contrast, when a RERI estimate is first calculated for each study and these study-level RERI estimates are meta-analyzed, the pooled RERI may not always match population-level risk estimates. This may have several reasons, but is at least partly due to the skewed distribution of RERIs that results from the exponentiation step in the algorithm used to calculate RERIs from the log-relative-risks (see article).

In this real-world data example we show how direct pooling of study-level RERI estimates in two-stage IPD meta-analysis leads to an estimate for the overall RERI that does not match the estimated effects of the exposures and their product term in the population. The data used in this example are derived from a meta-analysis of Keijzer et al. (2007) on the interaction between factor V Leiden and hyperhomocysteinemia in venous thrombosis. This meta-analysis involved five case-control studies. The results of these studies are provided in Table S1. In each study odds ratios (ORs) were used to calculate a study-level RERI estimate. Study-level RERI estimates were subsequently pooled using both fixed-effects meta-analysis, similar to the original meta-analysis, and random-effects meta-analysis, allowing effects to vary across studies. The results are provided in Table S2. To compare to what extend these pooled RERI estimates fit the estimated effects for both exposures and their product term in the population, we calculated RERI estimates based on the pooled individual effect estimates, following the procedure proposed in this article (Table S2). For both fixed-effects and random-effects meta-analysis, pooling of study-level RERIs resulted in a negative overall RERI (-1.77 and -0.48 respectively) suggesting negative interaction, while the overall RERI based on pooled effect estimates resulted in a positive RERI (2.47 and 1.94 respectively) suggesting positive interaction. Of note, confidence intervals for both procedures were rather large and one may conclude that there is little evidence of any additive interaction in this example. However, this is not the point of our demonstration, which is merely to show that the reported population-level RERI is at odds with the reported population-level effects, which seems undesirable no matter how precisely these are estimated.

Reference

Keijzer MB, Borm GF, Blom HJ, Bos GM, Rosendaal FR, den Heijer M. No interaction between factor V Leiden and hyperhomocysteinemia or MTHFR 677TT genotype in venous thrombosis. Results of a meta-analysis of published studies and a large case-only study. Thromb Haemost. 2007;97(1):32-7.

Table S1. Study-level odds ratios (OR) and RERI for the risk of venous thrombosis associated with factor V Leiden (*F*), hyperhomocysteinemia (*H*) and the combination of both factors, derived from the meta-analysis of Keijzer et al (2007).

Studies	Cases/ controls	Presence (+) or absence (+) of factor V Leiden (*F*) or hyperhomocystenemia (*H*)				OR_{F-H+} (*C* vs. *D*)	OR_{F+H-} (*B* vs. *D*)	OR_{F+H+} (*A* vs. *D*)	
		A (*F*+ *H*+)	B (*F*+ *H*-)	C (*F*- *H*+)	D (*F*- *H*-)	OR (95% CI)	OR (95% CI)	OR (95% CI)	RERI (95% CI)
Study 1	Controls	4	20	141	447	1.54	6.21	9.31	2.56
	Cases	6	20	35	72	(0.99; 2.41)	(3.18; 12.11)	(2.57; 33.81)	(-9.85; 14.98)
Study 2	Controls	0	5	6	110	3.48	3.90	8.35	1.98
	Cases	3	14	15	79	(1.29; 9.37)	(1.35; 11.27)	(0.41; 169)	(-23.57; 27.52)
Study 3	Controls	2	38	27	579	1.07	1.91	9.65	7.67
	Cases	4	15	6	120	(0.43; 2.65)	(1.02; 3.57)	(1.75; 53.29)	(-8.83; 24.17)
Study 4	Controls	1	30	22	408	2.95	5.21	22.88	15.72
	Cases	6	41	17	107	(1.51; 5.75)	(3.11; 8.74)	(2.73; 192)	(-32.91; 65.35)
Study 5	Controls	2	5	11	251	2.77	10.14	1.90	-10.01
	Cases	3	40	24	198	(1.32; 5.78)	(3.93; 26.18)	(0.32; 11.49)	(-20.43; 0.41)

Note. RERI = OR_{F+H+} - OR_{F+H-} - OR_{F-H+} + 1

Table S2. Overall RERI based on pooling study-level RERI estimates and overall RERI based on pooled effect estimates.

Meta-analysis	Pooled RERI from study-level RERIs (95% CI)	Overall RERI from pooled effect estimates[1] (95% CI)
Fixed-effects	-1.77 (-8.61; 5.08)	2.47 (-2.85; 7.79)
Random-effects	-0.48 (-9.53; 8.57)	1.94 (-5.05; 8.93)

Note. [1] Individual ORs (see Table S1) are first log transformed and subsequently pooled in multivariate meta-analysis.
